# Ferrimagnetic mPEG-*b*-PHEP copolymer micelles loaded with iron oxide nanocubes and emodin for enhanced magnetic hyperthermia–chemotherapy

**DOI:** 10.1093/nsr/nwz201

**Published:** 2020-01-17

**Authors:** Yonghong Song, Dongdong Li, Yang Lu, Kun Jiang, Yi Yang, Yunjun Xu, Liang Dong, Xu Yan, Daishun Ling, Xianzhu Yang, Shu-Hong Yu

**Affiliations:** 1 Key Laboratory of Advanced Catalytic Materials and Reaction Engineering, School of Chemistry and Chemical Engineering, Key Laboratory of Metabolism and Regulation for Major Diseases of Anhui Higher Education Institutes, Hefei University of Technology, Hefei 230009, China; 2 Institutes for Life Sciences, School of Medicine, South China University of Technology, Guangzhou 510006, China; 3 Division of Nanomaterials & Chemistry, Hefei National Laboratory for Physical Sciences at the Microscale, CAS Center for Excellence in Nanoscience, Hefei Science Center of CAS, Department of Chemistry, Institute of Biomimetic Materials & Chemistry, University of Science and Technology of China, Hefei 230026, China; 4 Zhejiang Province Key Laboratory of Anti-Cancer Drug Research, College of Pharmaceutical Sciences, Key Laboratory of Biomedical Engineering of the Ministry of Education, Zhejiang University, Hangzhou 310058, China

**Keywords:** magnetic hyperthermia therapy, sensitive thermal response, chemotherapy, magnetic targeting, theranostics

## Abstract

As a non-invasive therapeutic method without penetration-depth limitation, magnetic hyperthermia therapy (MHT) under alternating magnetic field (AMF) is a clinically promising thermal therapy. However, the poor heating conversion efficiency and lack of stimulus–response obstruct the clinical application of magnetofluid-mediated MHT. Here, we develop a ferrimagnetic polyethylene glycol-poly(2-hexoxy-2-oxo-1,3,2-dioxaphospholane) (mPEG-*b*-PHEP) copolymer micelle loaded with hydrophobic iron oxide nanocubes and emodin (denoted as EMM). Besides an enhanced magnetic resonance (MR) contrast ability (*r*_2_ = 271 mM^−1^ s^−1^) due to the high magnetization, the specific absorption rate (2518 W/g at 35 kA/m) and intrinsic loss power (6.5 nHm^2^/kg) of EMM are dozens of times higher than the clinically available iron oxide nanoagents (Feridex and Resovist), indicating the high heating conversion efficiency. Furthermore, this composite micelle with a flowable core exhibits a rapid response to magnetic hyperthermia, leading to an AMF-activated supersensitive drug release. With the high magnetic response, thermal sensitivity and magnetic targeting, this supersensitive ferrimagnetic nanocomposite realizes an above 70% tumor cell killing effect at an extremely low dosage (10 μg Fe/mL), and the tumors on mice are completely eliminated after the combined MHT–chemotherapy.

## INTRODUCTION

The magnetic nanoparticle (MNP) is a clinically available biomedical material due to both promising safety in the body and unique magnetism, and a series of iron oxide nanoparticle-based nanomaterials have been approved to serve as magnetic resonance (MR) contrast agents and iron replacement injection (such as ferumoxytol) [1–4]. Besides these well-known applications, MNPs can induce a heating effect in response to low-frequency radio waves [5,6], and the thermoresponsive TRPV1 calcium ion channel has been successfully activated by this magnetically induced heating using linked iron oxide nanoparticles and intracellularly encoded ferritin nanoparticles [7,8]. Moreover, magnetic hyperthermia could reach above 42°C in the MNP-accumulated local area to induce the cytotoxicity of tumor cells, which even caused thermal ablation when the local area was heated over 50°C under an AMF [9–11]. Recently, as a non-invasive therapeutic method, magnetic hyperthermia therapy (MHT) using magnetic fluids has been confirmed to be a promising thermal therapy against liver cancer [5], lung cancer [12] and prostate cancer [13]. Compared with traditional hyperthermia methods such as photothermal heating using near infrared irradiation, MHT performs remotely controllable heating under AMF without penetration-depth limit, which is particularly useful for the treatment of non-superficial tumors [14–16]. More importantly, MHT exhibits desirable potential in clinical treatment against cancer, and Jordan *et al*. designed and established a clinically available magnetic fluid hyperthermia therapy system. Then, the clinical trial of MHT was performed on the treatment of brain tumor for the first time, and two patients suffering from glioblastoma multiforme received magnetic fluid hyperthermia therapy [17]. Meanwhile, iron oxide nanocrystals approved by the US Food and Drug Administration in clinical diagnosis exhibited the magnetic hyperthermia property to kill cancer cells [18,19].

However, limited by the poor heating conversion efficiencies of MNPs under AMF, a high dose of MNPs is generally required for an appropriate heating efficacy around the tumor region [20,21]. The high dosage leads to high cost, and further causes some side effects including non-targeted deposition, which limits the practical application of MHT. Until now, many efforts have been made to improve the therapeutic efficacy of MNP-mediated MHT. First of all, via regulating the shape [22–24], size [25–27], exchange coupling [21,28], magnetic dopant [29,30] and surface modification [31,32], the specific absorption rate (SAR) value of MNPs could be enhanced significantly, which has been confirmed to contribute effectively to MHT. For instance, a series of magnetically exchange-coupled CoFe_2_O_4_@MnFe_2_O_4_ core–shell nanocomposites have been fabricated with tunable magnetocrystalline anisotropy, leading to a one order of magnitude increase of the specific loss power (SLP) than single-component MNP [21]. Moreover, the combination of MHT with chemotherapy [33], immunotherapies [34,35], photothermal ablation [36,37], or photodynamic therapy [38] could significantly promote the therapeutic efficiency of MHT against tumors due to the synergistic effect. With the development of magnetic navigation, magnetic targeting has been employed to accelerate and increase the accumulation of MNPs in tumor tissues, leading to higher magnetic hyperthermia efficacy and targeted local heating treatment [39]. Very recently it was revealed that MHT could significantly promote a killing effect in human neuroblastoma cells loaded with MNP cells in comparison with an exogenous heating protocol, indicating the necessity of targeted delivery of these thermal-induced magnetic nanoagents for MHT [40]. Thereby, the achievement of magnetic nanocarriers with both high magnetic response and thermal sensitivity is particularly crucial for the further development and clinical translation of MNP-mediated MHT.

**Figure 1.**
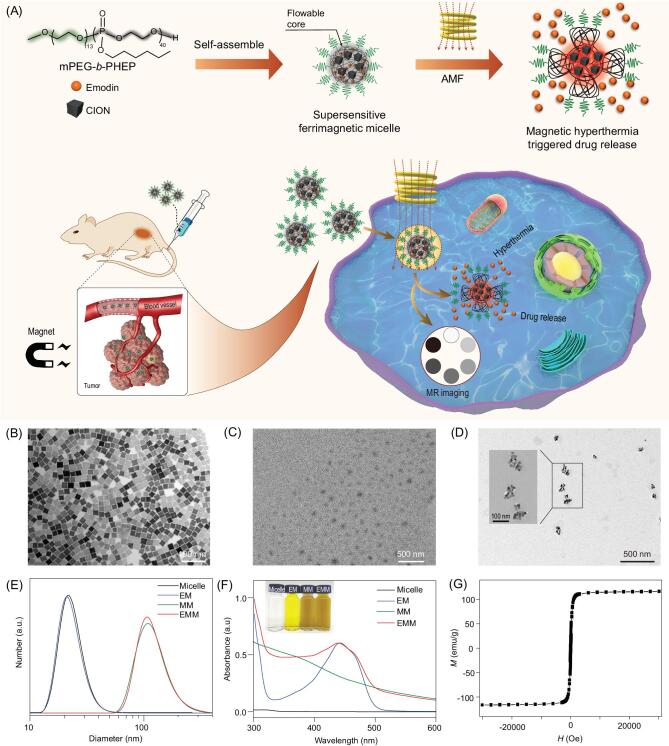
(A) Schematic illustration of the preparation of the emodin-loaded ferrimagnetic micelle (EMM) and magnetic targeting-guided cellular uptake. After exposure to AMF, the CION-loaded EMM induced magnetic hyperthermia therapy and rapid release of emodin. TEM images of (B) hydrophobic CION, (C) bare micelles and (D) EMMs. (E) The size distributions and (F) the ultraviolet visible spectra of the bare micelle, EM, MM and EMM. (G) Magnetic hysteresis curve of EMMs.

Here, we report a novel drug-loaded ferrimagnetic micelle with a flowable core to realize magnetic hyperthermia and chemotherapy synergistically. Cube-shaped ferrimagnetic iron oxide nanocrystals (CION) were selected as magnetic hyperthermia mediators that showed high magnetic performance for both MR diagnosis and magnetic therapy [41,42]. Emodin (3-methyl-1,6,8-trihydroxyanthraquinone) is the main active constituent extracted from the Chinese traditional herbal medicine *Rheum palmatum*. The antitumor activities of emodin have been discovered and confirmed on hepatocellular carcinoma, lung carcinoma and breast cancer [43,44]. However, its antitumor activity was restricted by its hydrophobic character. To promote the therapeutic effect of emodin and achieve an efficient magnetically induced heating-triggered release, we applied the amphiphilic block polymer mPEG-*b*-PHEP to load both CION and emodin as shown in Fig. 1A, which has successfully served as a nanocarrier with AMF-induced hyperthermia and supersensitive drug release [45,46]. As a magnetothermal responsive nanocarrier, the obtained emodin-loaded ferrimagnetic micelle (EMM) could be effectively taken up by tumor cells under the guidance of magnetic targeting. After exposure to AMF, the CION-induced temperature rise resulted in both the magnetic hyperthermia and supersensitive drug release to kill cancer cells simultaneously. Furthermore, this magnetic targeting nanocarriers served as a platform to combine with contrast-enhanced MR diagnosis against cancer.

## RESULTS AND DISCUSSION

### Preparation and characterization of emodin-loaded ferrimagnetic micelle

To fabricate the emodin-loaded ferrimagnetic micelle (EMM), we employed the amphiphilic polymer mPEG-*b*-PHEP (Supplementary Fig. 1A in the online supplementary material) as a nanocarrier to load hydrophobic drugs within its flowable hydrophobic PHEP core. Based on our previous report [46], the PHEP homopolymer possesses a much lower glass transition temperature (−81.8°C) than body and room temperatures (Supplementary Fig. 1B), so the PHEP chains in the hydrophobic core are in a unique flow state in the obtained micelle, which can realize the supersensitive drug release from the flowable PHEP core in response to slight heating stimuli. Here, we applied powerful ultrasonication to encapsulate both the antitumor drug emodin and oleic acid-coated CION into the mPEG-*b*-PHEP-based polymeric micelle. The transmission electron microscopy (TEM) image of hydrophobic CION showed the uniform cube-like shape in a diameter of about 22 nm (Fig. 1B). Compared with the monodispersed CION in chloroform, the obtained micelle was well dispersed in water. The TEM image of the bare micelle exhibited a spherical morphology with a diameter around 50 nm (Fig. 1C). In comparison, as shown in Fig. 1D, the EMM encapsulated several uniform

CION nanocubes in each micelle, and the diameter was around 100 nm. In the higher resolution TEM image (inserted image), the indistinct layer on the surface of the CION indicated the presence of a PEG-PHEP layer. Dynamic light scattering was applied to measure the hydrodynamic diameter of the bare micelle, emodin-loaded micelle (EM), ferrimagnetic micelle (MM) and EMM (Fig. 1E). Compared with the bare PHEP micelle and EM, the increased diameters of MM and EMM were matched with the observation in TEM images (Fig. 1B–D), which indicated the successful loading of CION in micelles. The concentrations of Fe and emodin were measured by inductively coupled plasma atomic emission spectroscopy (ICP-AES) and ultraviolet–visible (UV-Vis) absorption spectroscopy, respectively, and the encapsulation efficiency of CION and emodin were 62.0% ± 0.6% and 73.8% ± 2.8%, respectively. As shown in Fig. 1F, owing to the encapsulation of the active compound emodin into the micelle, EMM and EM solutions revealed an obvious UV-Vis absorption peak around 445 nm. The photographs of these micelle aqueous dispersions matched with the results of UV-Vis absorption spectra, confirming the stable encapsulation of hydrophobic CION and emodin in the mPEG-*b*-PHEP micelle. In addition, EMMs dispersed in de-ionized water (DIW) and culture medium containing 10% fetal bovine serum could maintain their hydrated diameters over 7 days, and the stability in hydrated diameter was attributed to the PEGylated modification (Supplementary Fig. 2B). Due to the effective loading of cubic-like iron oxide nanocrystals, the obtained EMM exhibited enhanced ferrimagnetism, and the *M*–*H* curve of EMM lyophilized powder is shown in Fig. 1G. The saturation magnetization value of EMM (116 emu/g(Fe)) was higher than most frequently used magnetite nanoparticles [47], which was nearly twice as high as the performance of the clinically available Resovist (65 emu/g(Fe)) [48]. The magnetic performance of MNPs plays a crucial role in their theranostic application [2], and such a high ferrimagnetism is reasonable to improve both *T*_2_*-*weighted MR diagnosis sensitivity and the magnetically induced heating ability.

### Magnetic hyperthermia performance of emodin-loaded ferrimagnetic micelle

**Figure 2.**
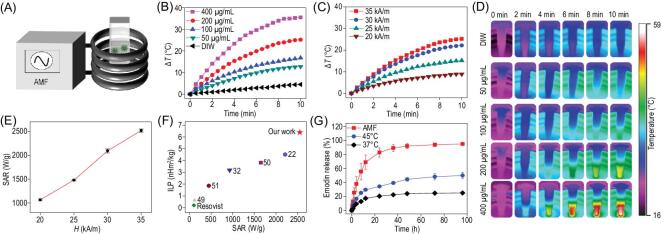
(A) Schematic illustration of the magnetic hyperthermia test. Time-dependent temperature change curves with (B) different EMM concentrations and (C) different AMF intensities. (D) Thermal images of (B). (E) The SAR values of EMMs at different AMF intensities. (F) The ILP and SAR values comparison of EMMs and other iron oxide nanomaterials. (G) The release curves of emodin from EMM dispersions after the treatment for 10 min at 37°C and 45°C, and in response to the stimuli under AMF.

To investigate the magnetically induced heating capability of the CION-loaded mPEG-*b*-PHEP micelle, we measured the hyperthermia performances under AMF with varying CION concentrations and magnetic field intensities. As illustrated in Fig. 2A, all samples at various concentrations in 1.5 mL tubes were located at the center of a copper coil. To test the iron concentration-dependent heating effect, MM samples (ranging from 50 to 400 μg/mL) were exposed to AMF at a magnetic field intensity (*H*) of 35 kA/m. As shown in Fig. 2B, it was found that 200 μg/mL of MM performed an excellent hyperthermia effect, in which the temperature rose about 24°C within 10 min. In addition, the magnetic field intensity (35, 30, 25 and 20 kA/m)-dependent heating effect was also revealed (Fig. 2C). The infrared thermal images of the MM dispersion with varying concentrations (Fig. 2D) further confirmed the magnetothermal heating performance. Specific absorption rate (SAR) and intrinsic loss power (ILP) were investigated to quantitatively evaluate the heating capability of MMs [22,30]. Figure 2E shows the SAR values of EMMs under the different intensities of AMF, and the largest SAR value reached 2518 W/g at 35 kA/m. In the same condition, the ILP was calculated to be 6.5 nHm^2^/kg (Fig. 2F). The SAR and ILP values of this ferrimagnetic micelle were dozens of times higher than the clinically available iron oxide nanoagents, such as Feridex (115 W/g and 0.16 nHm^2^/kg) and Resovist (104 W/g and 0.21 nHm^2^/kg) [21,22]. Such high SAR and ILP values for EMM also indicated the superior heating conversion efficiency in comparison with recently reported values of iron oxide nanomaterials [22,32,49–51].

As a drug delivery system, the mPEG-*b*-PHEP-based polymeric micelle with a flowable core exhibited supersensitive drug release under the near-infrared light heating stimuli, which has been confirmed in previous reports by our group [46]. Thus, the release of emodin loaded in this magnetic composite micelle can be accelerated after exposure to AMF (35 kA/m). As shown in Fig. 2G, only 20% of emodin was released from the EMM at 37°C, and the release rate was increased to 45% after the dispersion was pretreated at 45°C for 10 min. Furthermore, the loaded emodin was almost completely released from the EMM after exposure to AMF for 10 min, which should be attributed to the internal magnetically induced heating. These results suggested that the rapid emodin release could be triggered by a remote AMF using this ferrimagnetic composite micelle. In addition, poly(D,L-lactide) (PLA) is one of the frequently used biomaterials [46], and we prepared the PLA-*b*-PEG-based emodin magnetic micelle (EMM^PLA^) as a control sample without the flowable core. After the same treatment with AMF stimuli, as showed in Supplementary Fig. S3, the PLA-based magnetic micelle showed a much slower drug release speed owing to the rigid core, and the sensitive speed of the PHEP-based micelle demonstrated its high flowability.

### Enhanced uptake of EMMs under magnetic targeting

**Figure 3.**
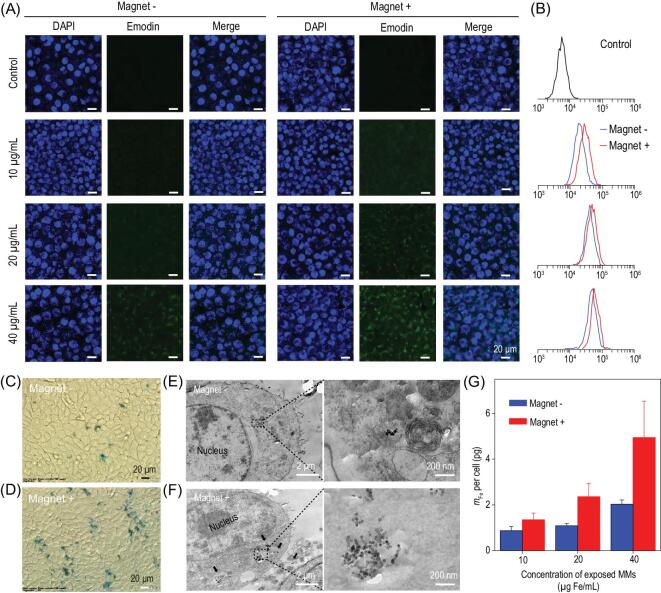
Cellular uptake of EMMs under magnetic targeting. (A) Confocal laser scanning microscopy images and (B) corresponding flow cytometry results of 4T1 cells exposed to EMMs at varying concentrations in the presence or absence of an external magnet. All scale bars are 20 μm. (C, D) Iron staining images and (E, F) cellular TEM images of 4T1 cells co-cultured with 40 μg/mL MMs. (G) The iron mass per cell measured by ICP-AES analysis after the exposure to MMs.

The magnetic targeting efficiency of EMMs was evaluated in 4T1 cells. As a natural active ingredient, emodin shows green fluorescence [52]. Thus, confocal laser scanning microscopy was used to explore the cell uptake of EMMs in the presence and absence of an external magnetic field. As shown in Fig. 3A, the cells were exposed to EMMs at different concentrations for 24 h, the cell nucleus was labeled by 4^′^,6-diamidino-2-phenylindole (DAPI) with blue fluorescence. The green fluorescence intensity in the cytoplasm was noticeably stronger at the higher concentration of EMMs, indicating that more emodin was taken up into 4T1 cells. Compared with the treatment without the magnet (Magnet− cells), there was stronger green fluorescence in the cell cytoplasm in the presence of the magnet (Magnet+ cells), which confirmed that the uptake efficiency could be elevated by magnetic targeting. Flow cytometry analysis was employed to further quantify the enhanced uptake of emodin in Magnet+ cells (Fig. 3B). Importantly, when the Magnet+ cells were exposed to 20 μg/mL EMMs, its fluorescence intensity was comparable to the Magnet− cells exposed to 40 μg/mL EMMs. This result indicated that only a half-dosage of antitumor drug was required to obtain the same chemotherapeutic effect under magnetic targeting. To investigate the cellular uptake of CION, 4T1 cells were exposed to 40 μg/mL MMs in the presence and absence of a magnet. After 24 h incubation, Prussian blue was employed to stain the iron oxide nanoparticles in MMs. As shown in Fig. 3C and D, in comparison with Magnet− cells, the image of Magnet+ cells showed more blue plots, indicating that the enhanced uptake of MMs was attributed to the magnetic targeting guidance. To visually observe the intracellular distribution of MMs, TEM was employed to explore the endocytosis route. As shown in Fig. 3E, the TEM images show internalized MMs in the cytoplasm and lysosomes of Magnet− cells, owing to the feasibility of mPEG-*b*-PHEP-based delivery. Compared with Magnet− cells, there were more MMs taken up in the cytoplasm observed in the Magnet+ cells (Fig. 3F), and the high-magnification TEM images confirmed the distinguished iron nanocubes. ICP-AES analysis was employed to further quantify the enhanced uptake of iron mass in cells under magnetic targeting, which has more than doubled in Magnet+ cells after incubation with MMs at the concentrations of 20 and 40 μg/mL (Fig. 3G). According to the previous report [40], this improvement of iron oxide cellular uptake under magnetic targeting should contribute to promoting the magnetic hyperthermia effect against cancer cells.

### Integration of magnetic hyperthermia–chemotherapy *in vitro*

**Figure 4.**
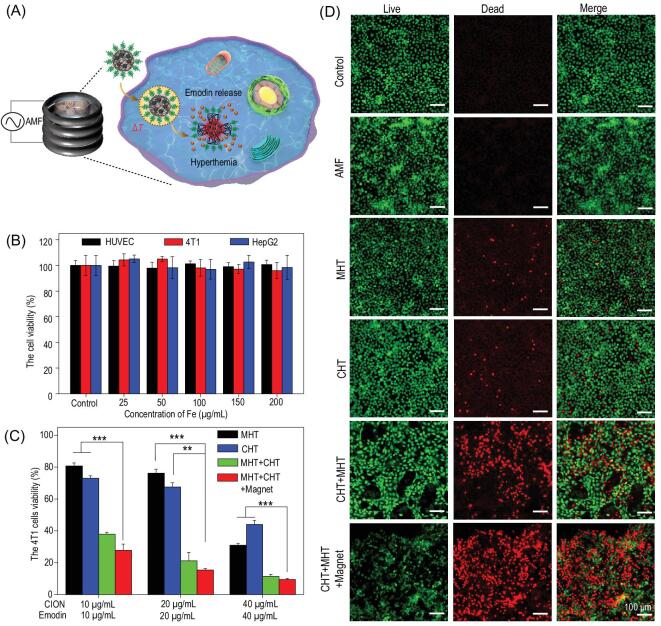
(A) Schematic illustration of EMM nanocomposites as therapeutic agents to accomplish magnetic hyperthermia–chemotherapy synergy therapy effect for 4T1 cells. (B) Viability of the 4T1 cells after incubation with MM nanocomposites for 24 h. (C) Viability of the 4T1 cells after exposure to MMs with AMF (MHT), EMMs with AMF (MHT+CHT) or not (CHT) and EMMs with AMF under magnetic targeting (MHT+CHT+Magnet) for 24 h. (D) The live–dead co-staining images of 4T1 cells in (C). All scale bars are 100 μm.

The magnetic hyperthermia and chemotherapy performance of the EMM was evaluated in 4T1 cells. As illustrated in Fig. 4A, after the cancer cells were co-incubated with EMMs in a culture dish, the EMM was effectively internalized in cells by magnetic targeting. After exposure to AMF, the release of emodin was accelerated owing to the abrupt magnetically induced heating inside the ferrimagnetic micelle, so magnetic hyperthermia and chemotherapy would be integrated to greatly improve the killing effect of the cells. Iron oxide nanomaterials have been widely used in the biomedical field, and their excellent stability and safety are the basic characters for clinical approval [53]. Thus, the standard CCK-8 assay was applied to evaluate the compatibility of MMs on normal (HUVEC) and cancer (4T1 and HepG2) cells. As shown in Fig. 4B, no obvious cellular toxicity was detected after incubation for 24 h, even when exposed to MMs at a high concentration (200 μg/mL). The 4T1 cancer cells were exposed to MMs following AMF treatment to evaluate the single magnetic hyperthermia therapy (MHT) effect, while exposure to EMMs was employed to evaluate the single chemotherapy (CHT) effect. As shown in Fig. 4C, both MHT and CHT treatments showed obvious dose-dependent cytotoxicity, and ∼20% or ∼25% cells were killed by MHT or CHT at a low dosage (10 μg/mL), respectively. In comparison, when the cells were exposed to EMMs with AMF (MHT+CHT), the cell death rate was up to 60%, so the combined therapeutic effect of MHT and CHT was superior to the sum of single treatments. Besides the sum of MHT and CHT, this synergistic enhancement was also attributed to the thermal-responsive block polymer micelle and excellent magnetic response, which accelerated the release of emodin from EMMs under the remote AMF stimuli. Moreover, magnetic targeting could further enhance the combined MHT+CHT therapeutic effect, and there was a more than 10% reduction of the cellular viability at a low concentration (10 μg/mL) (Fig. 4C). Therefore, an above 70% cell killing effect was achieved at an extremely low dosage of EMMs (10 μg Fe/mL), which was dozens of times less than the required amount of magnetic material in previous reports [19,22,30]. What is more, these targeting synergies were able to further reduce the dose of drugs, achieving a more efficient and safe cancer treatment. Live (calcein AM, green) and dead (propidium iodide (PI), red) double staining was employed to directly verify the synergetic therapy effect. As shown in Fig. 4D, there were numerous PI-stained dead cells in CHT+MHT, and fewer live cells could be observed in the CHT+MHT+Magnet group, which further demonstrated the synergetic therapy effect and magnetic targeting efficiency.

### *T*_2_-weighted MR imaging

**Figure 5.**
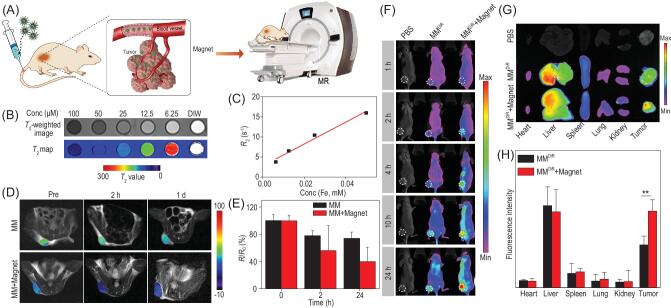
(A) Schematic illustration of EMM nanocomposites effectively accumulated at a tumor site under magnetic targeting for *T*_2_-weighted MR imaging. (B) The *T*_2_-weighted image, map and (C) the transverse relaxivity *r*_2_ of EMMs. (D) The *in vivo T*_2_-weighted images and (E) normalized *T*_2_ signal change of 4T1 tumor-bearing mice after intravenous injection of MM nanocomposites in the absence and presence of magnetic targeting. (F) The fluorescence images of 4T1 tumor-bearing mice after intravenous injection of DiR-loaded MMs (MM^DiR^) at 1, 2, 4, 10 and 24 h. (G) Fluorescence images and (H) quantitative results of DiR fluorescence intensity in major organs and tumor.

Because of the remarkable saturation magnetization of EMMs, the expectant perspective of the EMM to be treated as a *T*_2_ MR imaging contrast was indicated in Fig. 5A [42,54]. Specifically, we collected the *T*_2_-weighted image *in vitro* as shown in Fig. 5B. It can be seen that the MR signal intensity was clearly enhanced as the concentration of EMMs decreased. According to the curve in Fig. 5C, the transverse relaxivity (*r*_2_) of EMM dispersion was calculated to be as high as 271 mM^−1^ s^−1^, which indicated the excellent MR imaging performance for cancer diagnosis. The enhanced permeability and retention (EPR) effect of mPEG-*b*-PHEP nanocarriers is illustrated in Fig. 5A [45]. We further examined the magnetic targeting feasibility of MR imaging towards the 4T1 tumor-bearing mouse by intravenous injection of MM dispersion (10 mg/kg). As shown in Fig. 5D, in *vivo* MR images showed that the *T*_2_ intensity of the MM+Magnet group was markedly decreased compared with that in the MM group. Significant persistence of the hypo-signal was observed in the MM+Magnet group after 24 h of injection, and the quantitative relaxation rates (*R*_2_/*R*_0_) showed a reduction of 60.1% in the MM+Magnet group, while there was only 26.1% reduction in the MM group (Fig. 5E). We also collected the tumors from the mice in each group after treatment. As shown in Supplementary Fig. 4, the blue plots indicated the stained iron oxide in EMMs. By comparison, the stained regions in the MM+Magnet group were obviously more than those in the MM group. This enhanced accumulation of iron oxide nanocrystal-loaded micelles in the tumor should be attributed to efficient magnetic targeting.

### *In vivo* biodistribution

The MMs labeled with 1,1-dioctadecyl-3,3,3,3-tetramethylindotricarbocyanine iodide (DiR) were applied to investigate the distribution of our micelles in 4T1 tumor-bearing mice. After intravenous injection of MM^DiR^ into the mice, the fluorescence images were observed by the In-Vivo Xtreme system. As shown in Fig. 5F, in the mouse without magnet treatment, the fluorescence intensity of DiR was gradually enhanced in the tumor site within 10 h and was almost retained after 24 h. By contrast with magnet treatment, the evident high accumulation of fluorescence intensity in the tumor appeared to be due to the efficient magnetic targeting—even after 24 h, the fluorescence signal still increased. Furthermore, the mice were sacrificed at 24 h post-injection and the major organs and tumor tissue were harvested from the mice for further imaging. As shown in Fig. 5G, both groups without-magnet and with-magnet showed weak fluorescence intensity in heart,

lung, spleen and kidney, but an obvious distribution in liver. Besides, both the tumor tissue images and the corresponding quantitative fluorescence intensity results demonstrated our nanocarriers, with the help of an external magnetic field, had an outstanding EPR effect. Furthermore, all the data suggested the advantage of magnetic targeting-guided high accumulation of EMMs in the tumor region.

### Integration of magnetic hyperthermia–chemotherapy *in vivo*

**Figure 6.**
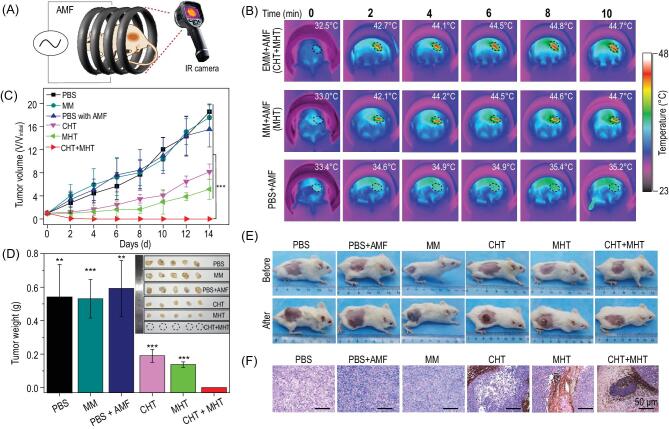
(A) Schematic illustration of EMM-mediated magnetic hyperthermia–chemotherapy *in vivo*. (B) Infrared thermal images of mice with the administration of PBS, MMs or EMMs under AMF (*H* = 30 kA/m, *f* = 312 kHz) for 10 min. (C) The 4T1 tumor growth curves and (D) mice tumor weight of six groups after receiving different treatments. (E) Digital images of mice from each group before and after treatment. (F) The H&E staining of tumor tissues from each group after treatment. All scale bars are 50 μm. Data are presented as mean ± SD, *n* = 5.

Consistent with the remarkable *in vitro* results, we further examined the *in vivo* magnetic hyperthermia–chemotherapy effect on the 4T1 tumor-bearing mice. As illustrated in Fig. 6A, after intratumoral injection of EMM solution into the tumor site of the mouse, the mouse was placed into the magnetic induction coil and exposed to an AMF (30 kA/m, 312 kHz) for 10 min. As observed in the infrared thermal images (Fig. 6B), compared with the injection of phosphate-buffered saline (PBS), the temperature in the tumor region rose quickly after injection of EMM or MM dispersion, reaching 42°C within 2 min (Supplementary Fig. 5). This heating effect was enough to suppress tumor growth. As shown in Fig. 6C, compared with those control groups, the tumor volume of mice in the MHT group and the CHT group was significantly smaller, indicating single MHT or CHT treatment could inhibit the tumor growth, but neither of them could cure the tumor completely. However, the incorporation of both MHT and CHT exhibited pronounced tumor inhibition, and recurrence of the tumor was prevented. In addition, there was no significant body weight variation in any group after treatment (Supplementary Fig. 6). To visually observe these results, the photographs of mice and the excised tumor in each group before and 2 weeks after treatment are shown in Fig. 6D and E. Compared with the markedly enlarged tumors of mice in control groups, both CHT and MHT groups showed obvious inhibition of tumor. However, after MHT+CHT treatment, the tumor was completely eliminated. The hematoxylin & eosin (H&E) staining of tumors excised from mice in each group is shown in Fig. 6F and the tumor cells retained their original morphology without obvious necrosis in control groups. However, necrosis could be observed after treatment of the CHT or MHT only groups, and the cancer cells were completely destroyed throughout the tumor after

magnetic hyperthermia–chemotherapy, indicating the combined therapy resulted in more severe damage to cancer cells.

Furthermore, the evaluation of biocompatibility and biosafety of nanocarriers is a key procedure for further clinical application [55,56]. Here, EMMs (10 mg/kg) were injected intravenously into healthy ICR mice. Injection of the same dose of PBS was treated as control (*n* = 5). The blood samples were collected at 3 and 10 days after injection. As expected, all the values of the main biochemical parameters (Supplementary Fig. 7A) in the serum of mice were almost consistent in a normal range. In addition, the H&E staining of the main organs of the mice also clarified that there was no obvious difference between the mice of the control group and the mice injected with micelles (Supplementary Fig. 7B). Combined with these exciting results, our EMM showed good biocompatibility. Moreover, after injection of EMMs (10 mg/kg) under the AMF exposure, we studied the main liver and kidney function indices. As shown in Supplementary Fig. 8, compared with the control group, all the indices of the experimental group were in the normal range, indicating the safety of the EMM.

## CONCLUSION

In summary, a new kind of ferrimagnetic therapeutic nanocarrier with a flowable core can be fabricated here with excellent MR diagnosis effect, and magnetic hyperthermia performance, which further realizes the tumor-targeted drug delivery of the hydrophobic antitumor drug emodin and remotely controlled release in response to magnetic hyperthermia. The integration of magnetic hyperthermia and enhanced chemotherapy enables the complete killing of cancer cells at a low dosage of iron oxide nanocrystals and antitumor drugs, which should be meaningful to the reduction of the risk of side effects in the treatment of cancer as well as the clinical promotion of MHT–chemotherapy. Moreover, this magnetic hyperthermia–chemotherapy design with emodin could be extended to many other hydrophobic active ingredients extracted from natural products, such as triptolide and paclitaxel for the treatment of cancers including hepatocellular cancer and ovarian cancer.

## METHODS

### Fabrication of the diblock copolymer mPEG-*b*-PHEP and emodin-loaded ferrimagnetic micelle

Emodin and dimethyl sulfoxide (DMSO) were obtained from Sangon Biotech Co. Ltd. Trypsin, Dulbecco's modified Eagle medium **(**DMEM), fetal bovine serum and phosphate-buffered saline (PBS) were purchased from Gibco. Cell Counting Kit-8 (CCK-8) was purchased from Biosharp Co. Ltd. Iron (III) acetylacetonate (99%), 4-biphenylcarboxylic acid (95%), oleic acid (90%) and benzyl ether (98%) were purchased from Sigma-Aldrich. Methanol and chloroform were purchased from Sinopharm Chemical Reagent. The diblock copolymer mPEG-*b*-PHEP was synthesized according to our previous report by a ring-opening polymerization of the cyclic phosphoester monomer HEP [46]. Typically, 0.20 mmol of PEG (mol wt = 5000) and 11.7 mmol of HEP were added to 12 mL of anhydrous tetrahydrofuran, followed by the addition of 0.20 mmol of 1,5,7-Triazabicylo[4.4.0]dec-5-ene (TBD). After stirring for 6 min, the reaction was terminated by 2.0 mmol of benzoic acid to obtain the final product.

Cube-shaped ferrimagnetic iron oxide nanocrystal (CION) was synthesized as described in a previous report [57]. In brief, the benzyl ether (10.40 g) mixture containing iron (III) acetylacetonate (0.706 g), 4-biphenylcarboxylic acid (0.4 g) and oleic acid (1.129 g) was degassed for 2 h; it was then heated to 290°C and maintained for 30 min. Then the solution was washed twice with methanol and the obtained separated precipitate was CION. Typically, 20 mg mPEG-*b*-PHEP, 1 mg emodin and 1 mg CION were mixed in 2 mL tetrahydrofuran to prepare the emodin-loaded ferrimagnetic micelles. Then the mixture was dropped slowly into 10 mL DIW under ultrasound for 30 min. After the removal of tetrahydrofuran by vacuum rotatory evaporation, the free emodin was further removed by filter with a pore size of 450 nm. The obtained emodin-loaded ferrimagnetic micelle was then stored in a refrigerator at 4°C. To measure the concentration of emodin in EMMs, the aqueous dispersion was dissolved in methanol, and then the absorption value of the UV-Vis spectrum was tested at 449 nm. The concentration of emodin could be calculated by the standard curve of emodin.

### Characterizations

The morphology and structure were determined by transmission electron microscope (TEM, Hitachi HT7700). UV-Vis spectroscopy was performed on a UV-2600 (SHIMADZU, Kyoto, Japan). The magnetic hyperthermia effect was performed on high frequency (312 kHz) heating equipment (Shuangping SPG, China).

### Measurement of magnetic heating effect

To evaluate the magnetically induced heating effect of EMMs, EMM solutions with varying concentrations were placed inside the center of the copper coil. Under an alternating magnetic field (AMF) with a frequency of 312 kHz, the temperature changes of samples were recorded by an infrared thermography camera (Fluke, Ti400), and the specific absorption rate (SAR) and intrinsic loss power (ILP) were calculated by the following equations:

$$\begin{equation*}
{\mathit {SAR}} = C\frac{{\Delta T}}{{\Delta t}}\frac{1}{{{m_{Fe}}}},
\end{equation*}$$

$$\begin{equation*}
{\mathit {ILP}} = \frac{{SAR}}{{{H_2}f}},
\end{equation*}$$

where *C* is the specific heat capacity of the water, *m*_Fe_ represents the mass of the iron, Δ*T*/Δ*t* is the initial slope of the curve that shows the temperature–time change. *f* and *H* are the magnetic frequency and field intensity, respectively [21].

### Magnetic heating-induced drug release

A 1 mL EMM (300 μg (CION)/mL and 300 μg (emodin)/mL) solution was exposed to AMF (35 kA/m, 312 kHz). After 10 min AMF treatment, the sample was transferred into a dialysis bag (MWCO 3500 Da) and immersed in 20 mL PBS buffer (0.01 M, pH 7.4) with mild shaking (100 rpm, 37°C). The samples were exposed to AMF at predetermined times (0.5, 1, 2, 4, 8, 12, 24, 36, 48, 72 and 96 h). The collected solutions were freeze-dried and dissolved by 2 mL methanol to detect the concentration of emodin by UV-2600. In addition, EMM solutions were pre-incubated in a 45°C and a 37°C bath for 10 min at the same predetermined times, respectively, and their emodin release results were determined by the above method.

### Cell uptake study

4T1 cancerous cells, human umbilical vein endothelial cells (HUVECs) and HepG2 cells were obtained from the School of Life Science in the University of Science and Technology of China. To compare the cellular uptake efficiency of EMMs in the presence and absence of the guidance of magnetic targeting, 4T1 cells were exposed to EMMs at varying concentrations. The uptake of emodin was investigated by flow cytometry and a confocal laser scanning microscope (Carl Zeiss Jena, Germany) after 24 h of incubation. The uptake of the ferrimagnetic micelle iron was evaluated by the standard iron staining. After 24 h of being co-cultured with MMs, the cells were washed three times with PBS to remove free MMs. Then the cells were stained by potassium ferrocyanide (10 mg/mL dispersed in 1% hydrochloric acid) at 37°C for 20 min for further observation.

### *In vitro* biosafety

HUVEC, 4T1 and HepG2 cells, seeded into 96-well plates, were employed to evaluate the biosafety of our ferrimagnetic micelle. After 24 h in an incubator, the cells were exposed to MMs with varying concentrations of 25, 50, 100, 150 and 200 μg/mL. After being co-cultured for 24 h, 100 μL 1 mg/mL CCK-8 was added into every well and co-cultured for another 4 h. Finally, the plates were shaken for 10 min and the absorbance values of each well at 450 nm were measured by microplate reader (Thermo, Multiscan).

### *In vitro* magnetic hyperthermia and chemotherapy

To evaluate the therapeutic efficacy of EMMs, 4T1 cells were divided into four groups: (1) MHT group, the cells were exposed to MMs (10, 20, 40 μg/mL) with an AMF treatment; (2) CHT group, the cells were exposed to EMMs (10, 20, 40 μg/mL Fe, the corresponding emodin concentration was 10, 20, 40 μg/mL); (3) CHT+MHT group, the CHT group followed by AMF treatment; (4) CHT+MHT+Magnet group, based on the CHT+MHT group with magnetic targeting. After being co-cultured for 24 h, these groups with AMF treatment (312 kHz, 35 kA/m) were placed into a copper coil. After continuous heating for 10 min, all cells were incubated for another 24 h. Then the cells were cultured with 3-(4,5-dimethyl-2-thiazolyl)-2,5-diphenyl-2-H-tetrazolium bromide (MTT) for another 4 h, followed by 100 μL DMSO to dissolve the formazan. Finally, the absorbance value of supernatant was calculated by a microplate reader. For intuitive observation, calcein AM and propidium iodide (PI) were employed for live–dead double staining, and these results were observed on a fluorescence microscope (IX81, Olympus).

### Magnetic resonance imaging

The magnetic resonance imaging (MRI) performance of prepared EMMs was measured by a clinical 3.0 T MR scanner (Siemens, Germany). The measured parameters were as follows: repetition time (TR): 5000 ms, echo time (TE): 40–103 ms, FOV: 240 × 240 mm^2^. Then the *T*_2_-weighted MR images of samples at different concentrations (100, 50, 25, 12.5, 6.25 μM) were observed. The *T*_2_ value, obtained from images, was used to calculate the *R*_2_ (*R*_2_* *= 1/*T*_2_). Finally, the relaxivity coefficient (*r*_2_) value of the EMM solution was the slope of the linear fitting of the *R*_2_ value curve.

*T*_2_-weighted MR imaging on the 4T1 tumor-bearing mouse was performed to further evaluate the magnetic targeting effect. A small animal phased array coil (Suzhou Medcoil Healthcare Co., Ltd) was equipped on the same MR scanner with that *in vitro*. After the mice were intravenously injected by MM (10 mg/kg) solution, one group was exposed to a magnet placed near the tumor region. Then the *T*_2_-weighted axial images of mice were collected at 2 and 24 h after injection. The relaxation times of the tumor region were applied to evaluate the magnetic targeting MR imaging efficacy quantitatively.

### *In vivo* biodistribution study

To investigate the biodistribution of the EMMs, we loaded the 1,1-dioctadecyl-3,3,3,3-tetramethylindotricarbocyanine iodide (DiR) into the ferrimagnetic micelles (MM^DiR^). Then the 4T1 tumor-bearing mice were divided into three groups randomly: PBS group, without magnet group and with magnet group. PBS or MM^DiR^ (10 mg/kg Fe) was intravenously injected into each mouse. At the predetermined time points, the images of fluorescence biodistribution in mice were observed by In-Vivo Xtreme II (Bruker, Deutschland). The mice were sacrificed at 24 h post-injection and the major organs (heart, liver, spleen, lung, kidney and tumor) were collected for analysis by In-Vivo Xtreme II.

### *In vivo* magnetic hyperthermia and chemotherapy

For the construction of 4T1 tumor-bearing mice, Balb/c female mice (6–8 weeks old) supplied from Anhui Medical University received a subcutaneous injection of 4T1 cells (1.0 × 10^6^ per mice), and the tumor volume was calculated according to the followed equation: (tumor length) × (tumor width)^2^/2. When the tumor volume reached approximately 100 mm^3^, these tumor-bearing mice were divided to six groups (*n* = 5): (1) PBS group (100 μL), (2) PBS (100 μL) + AMF treatment group, (3) MM (5 mg (Fe)/kg bw), (4) MM (5 mg (Fe)/kg bw) with AMF (MHT) group, (5) EMM (5 mg (emodin)/kg bw + 5 mg (Fe)/kg bw) (CHT) group and (6) EMM (5 mg (emodin)/kg bw + 5 mg (Fe)/kg bw) plus AMF (MHT+CHT) group. After directly injecting these agents into the tumors of mice, a 10 min AMF treatment of *H* = 30 kA/m with *f* = 312 kHz was applied to each mouse. In this MHT process, the temperature changes of mice were recorded by an infrared thermography camera (Fluke, Ti400). After the treatment, the body weight and tumor volume of each mouse were recorded for 2 weeks. All animal experiments were performed in accordance with the recommendations in the *Guide for the Care and Use of Laboratory Animals* of the National Institutes of Health, which were approved by the Institutional Animal Care and Use Committee (IACUC) of Hefei University of Technology and Anhui Medical University (LLSC20150134).

### Statistical analysis

To measure significant differences among the treatment groups, statistical analyses were performed using Student's t-test. ^*^P < 0.05 was considered to be statistically significant, ^**^P < 0.01 and ^***^P < 0.001 were considered to be highly significant.

## Supplementary Material

nwz201_Supplemental_File
