# Supplementary material for: Ferrimagnetic mPEG-b-PHEP copolymer micelles loaded with iron oxide nanocubes and emodin for enhanced magnetic hyperthermia–chemotherapy
Source: Natl Sci Rev. 2020 Jan 17;7(4):723–36. doi: 10.1093/nsr/nwz201 (PMC8289054; doi:10.1093/nsr/nwz201)
Supplement: nwz201_Supplemental_File [file nwz201_supplemental_file.docx]

**Supplementary Information**

**Ferrimagnetic mPEG-*b*-PHEP Copolymer Micelles Loaded with Iron Oxide Nanocubes and Emodin for Enhanced Magnetic Hyperthermia-Chemotherapy**

Yonghong Song^1,🟉^, Dongdong Li^2,🟉^, Yang Lu^1,*^, Kun Jiang^1^, Yi Yang^1^, Yunjun Xu^3^, Liang Dong^3^, Xu Yan^1^, Daishun Ling^4^, Xianzhu Yang^2,*^, and Shu-Hong Yu^3,^^*^

^1^ Key Laboratory of Advanced Catalytic Materials and Reaction Engineering, School of Chemistry and Chemical Engineering, Key Laboratory of Metabolism and Regulation for Major Diseases of Anhui Higher Education Institutes, Hefei University of Technology, Hefei, Anhui 230009, P. R. China.

^2^ Institutes for Life Sciences, School of Medicine, South China University of Technology, Guangzhou, Guangdong 510006, China.

^3^ Division of Nanomaterials & Chemistry, Hefei National Laboratory for Physical Sciences at the Microscale, CAS Center for Excellence in Nano science, Hefei Science Center of CAS, Department of Chemistry, Institute of Biomimetic Materials & Chemistry, University of Science and Technology of China, Hefei 230026, China.

^4^ Zhejiang Province Key Laboratory of Anti-Cancer Drug Research, College of Pharmaceutical Sciences, Key Laboratory of Biomedical Engineering of the Ministry of Education, Zhejiang University, Hangzhou, 310058, PR China.

^🟉^ These authors contributed equally to this work.

^*^ Correspondence and requests for materials should be addressed to the author:

Yang Lu

Email: yanglu@hfut.edu.cn

Xianzhu Yang

Email: [yangxz@scut.edu.cn](mailto:yangxz@scut.edu.cn)

Shu-Hong Yu

Email: [shyu@ustc.edu.cn](mailto:shyu@ustc.edu.cn), Tel: 86-551-63603040, Fax: 86-551-63603040

**Supplementary Figures**


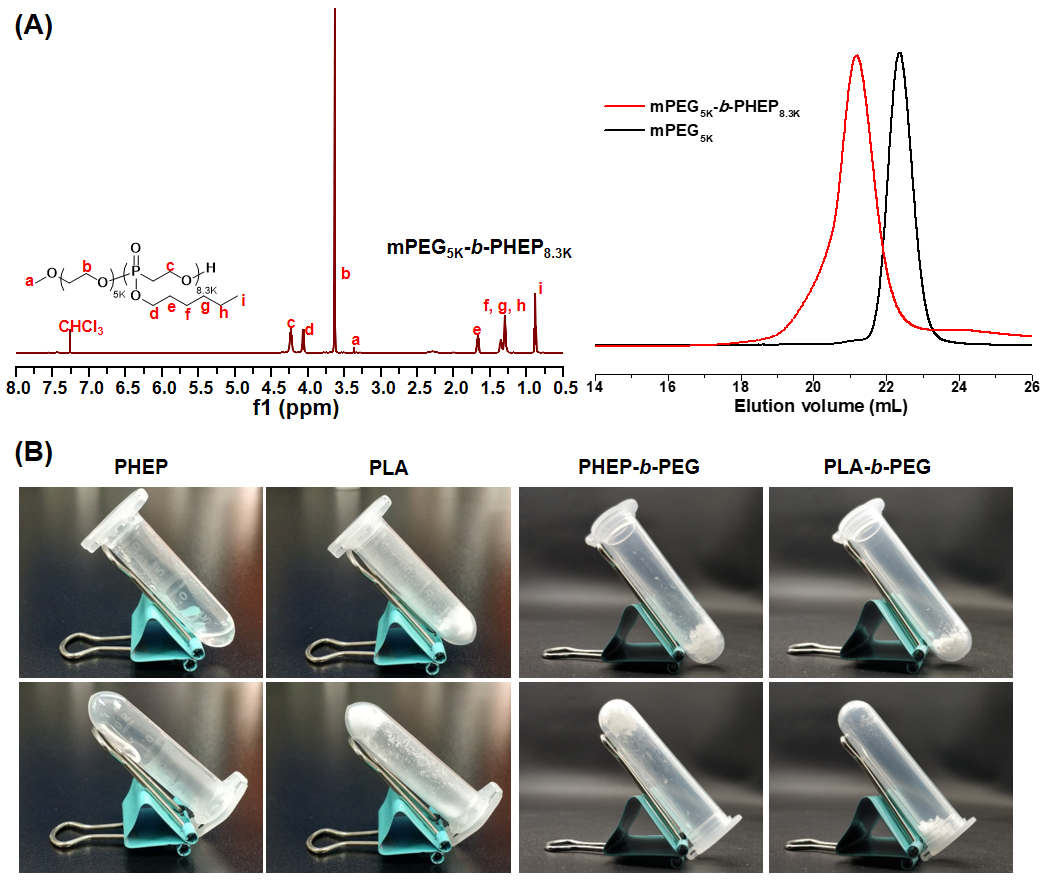


**Supplementary Fig. 1.** (A) ^1^H-NMR and GPC spectra of mPEG-*b*-PHEP. (B) The photo of PHEP, PLA, mPEG-b-PHEP and mPEG-*b*-PLA samples at room temperature.


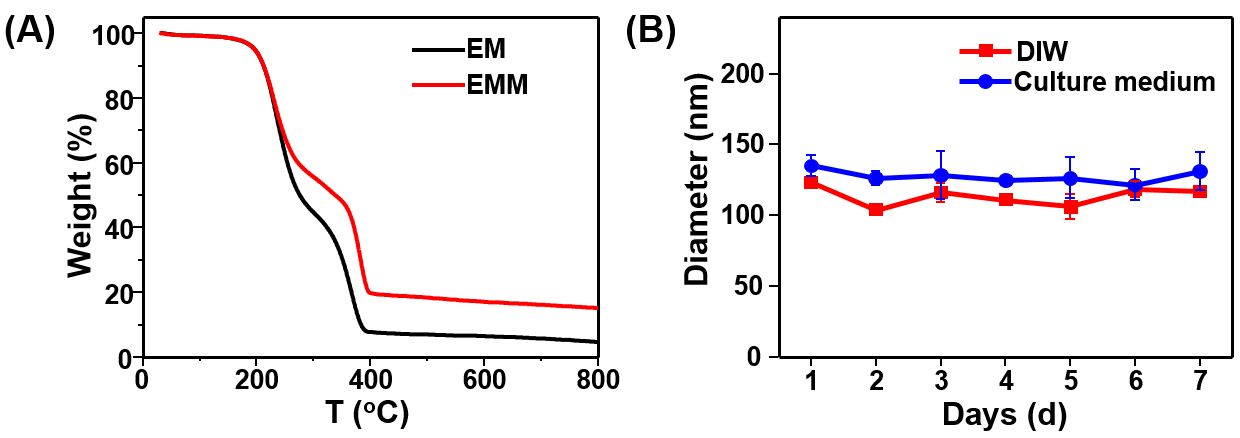


**Supplementary Fig. 2.** (A) TG analysis of EM and EMM nanocomposites. (B) The diameter variation of EMM dispersed in DIW and culture medium containing 10% FBS in 7 days. It clearly showed the excellent size stability.


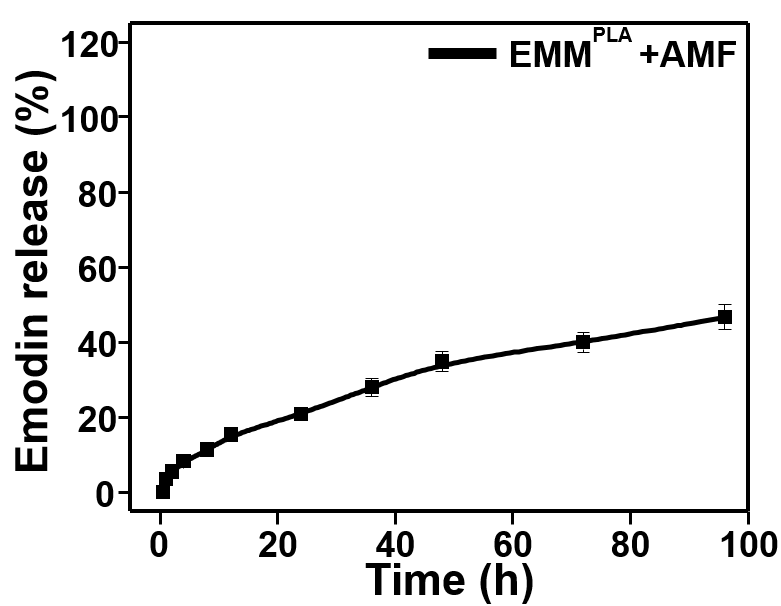


**Supplementary Fig. 3.** The emodin release curve of PLA based emodin magnetic micelle (EMM^PLA^) under the same AMF stimuli.


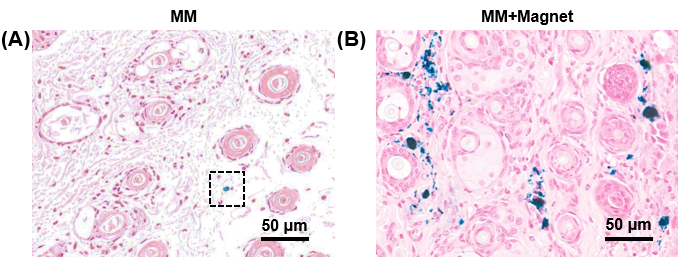


**Supplementary Fig. 4.** The iron staining images of tumor from mice in (A) MM group and (B) MM+Magnet group after the administration of MM. The blue plots indicated the stained iron oxide nanoparticles by Prussian blue, corresponding to the accumulation of CION loaded micelle in the tumor region.


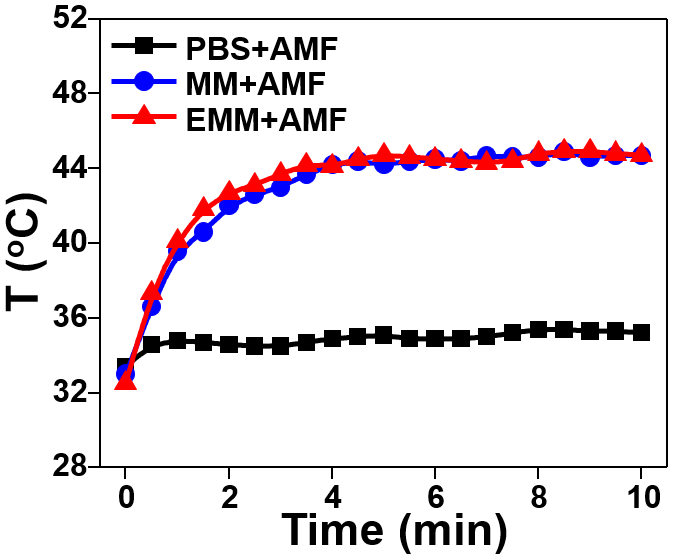


**Supplementary Fig. 5.** The temperature curves of the tumor sites under AMF treatment for 10 min. After injection of MM or EMM solution, the temperature in the tumor region rose quickly and reached 45 ^o^C within 2 minutes. In contrast, the temperature only reached to about 34-35 ^o^C after the injection of PBS.


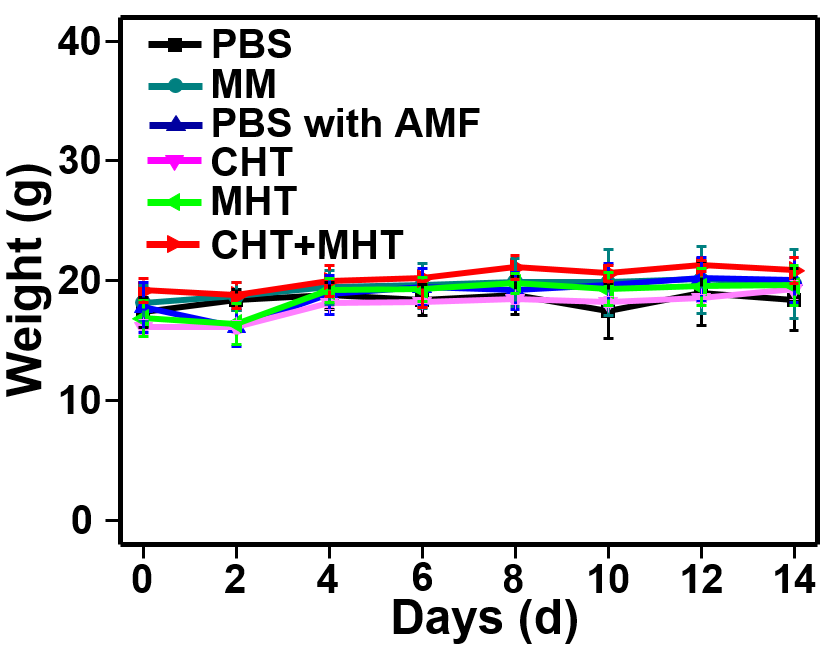


**Supplementary Fig. 6.** The 4T1 tumor-bearing mice body weight of six groups after receiving different treatment.


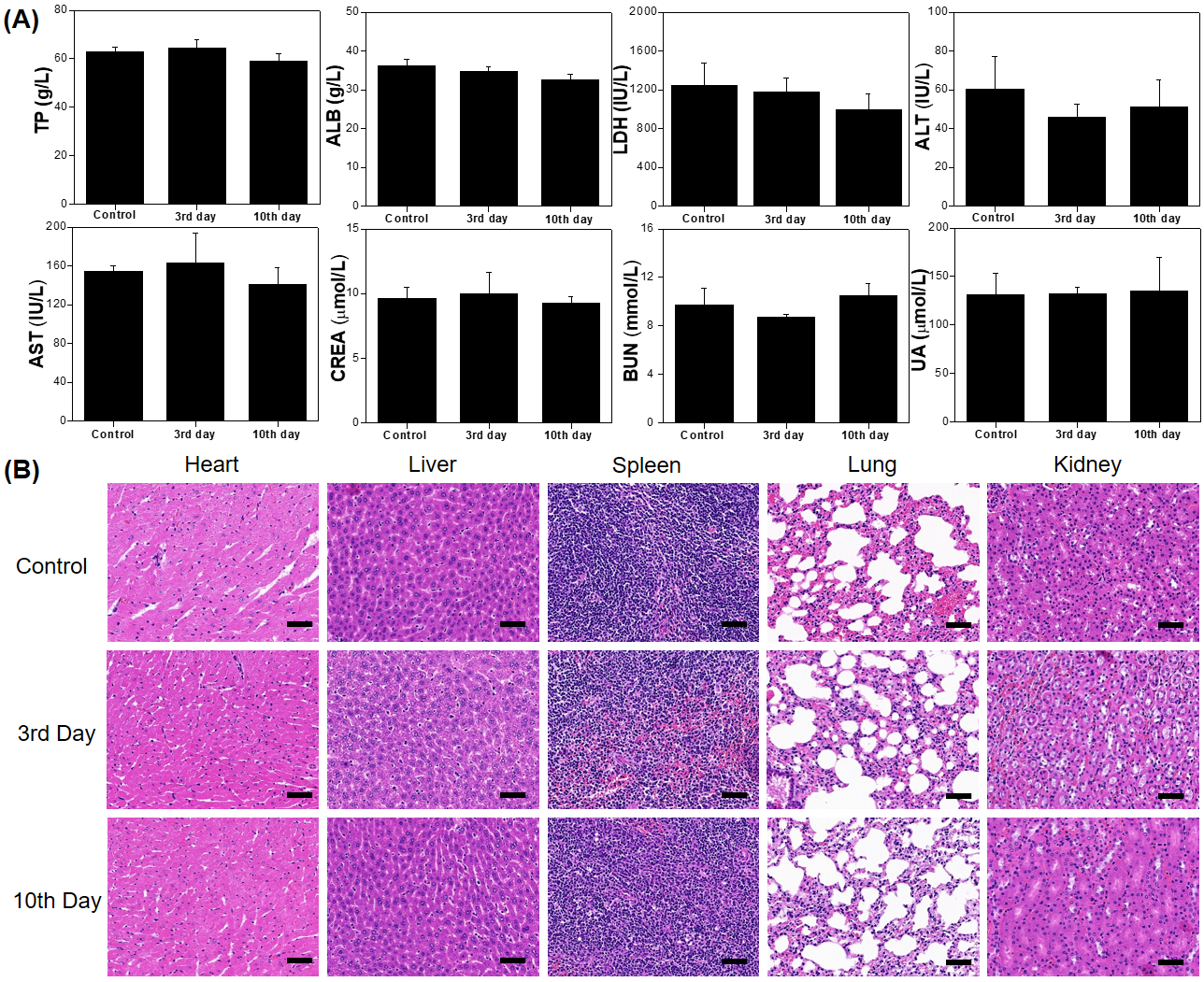


**Supplementary Fig. 7.** (A) Biochemical indices and (B) hematoxylin & eosin (H&E) staining images of major organs of mice at 3rd and 10th days after intravenous injection of EMM (10 mg/kg). Obviously, all values of biochemical indices of mice were almost consistent in a normal range. And no obvious pathological difference was clarified in the H&E staining images of heart, liver, spleen, lung and kidney between the normal and EMM administered mice. All scale bars are 50 μm.


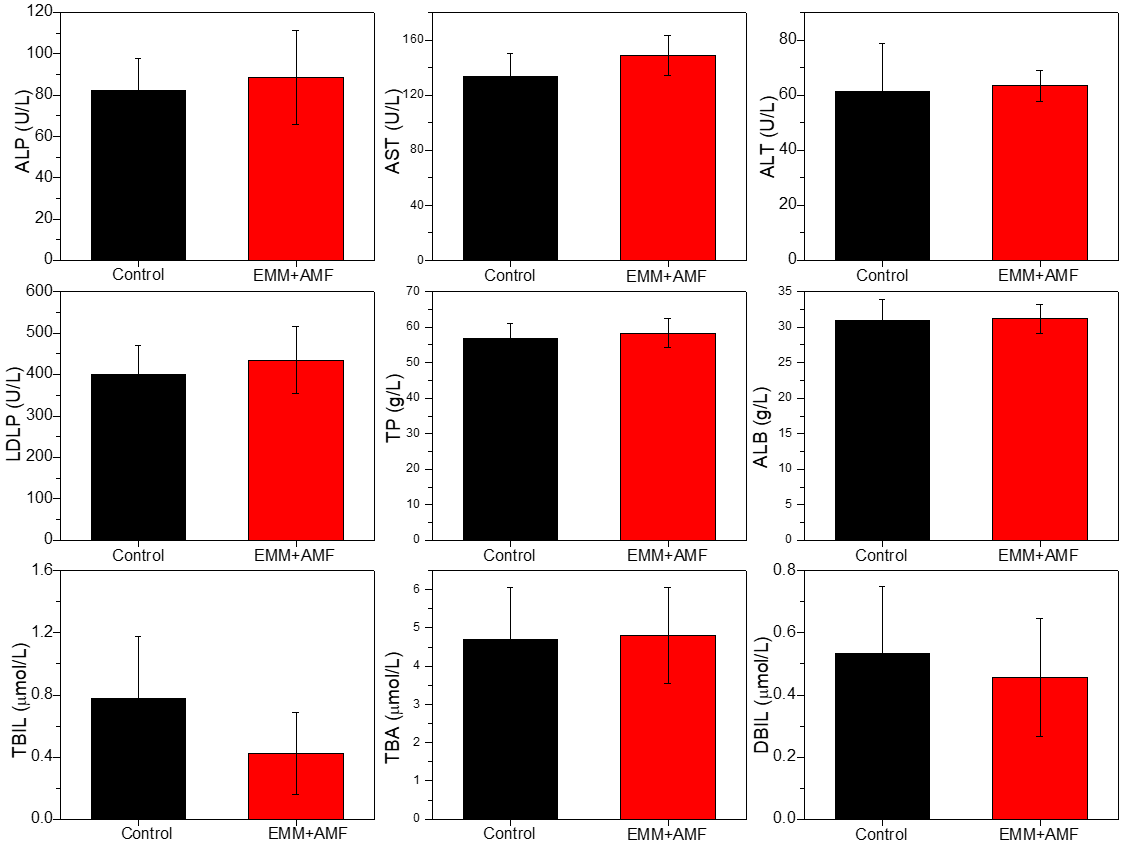


**Supplementary Fig. 8.** The main liver and kidney function indexes after injection of EMM (10 mg/kg) under the AMF exposure.
